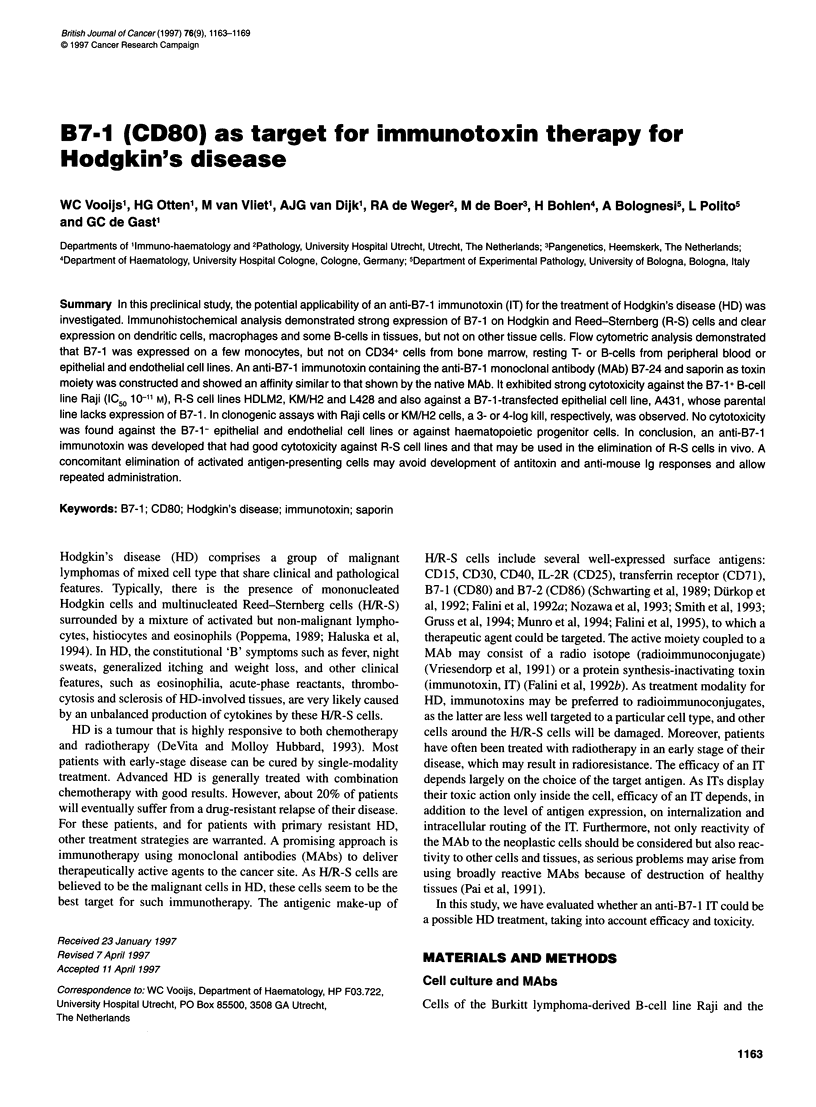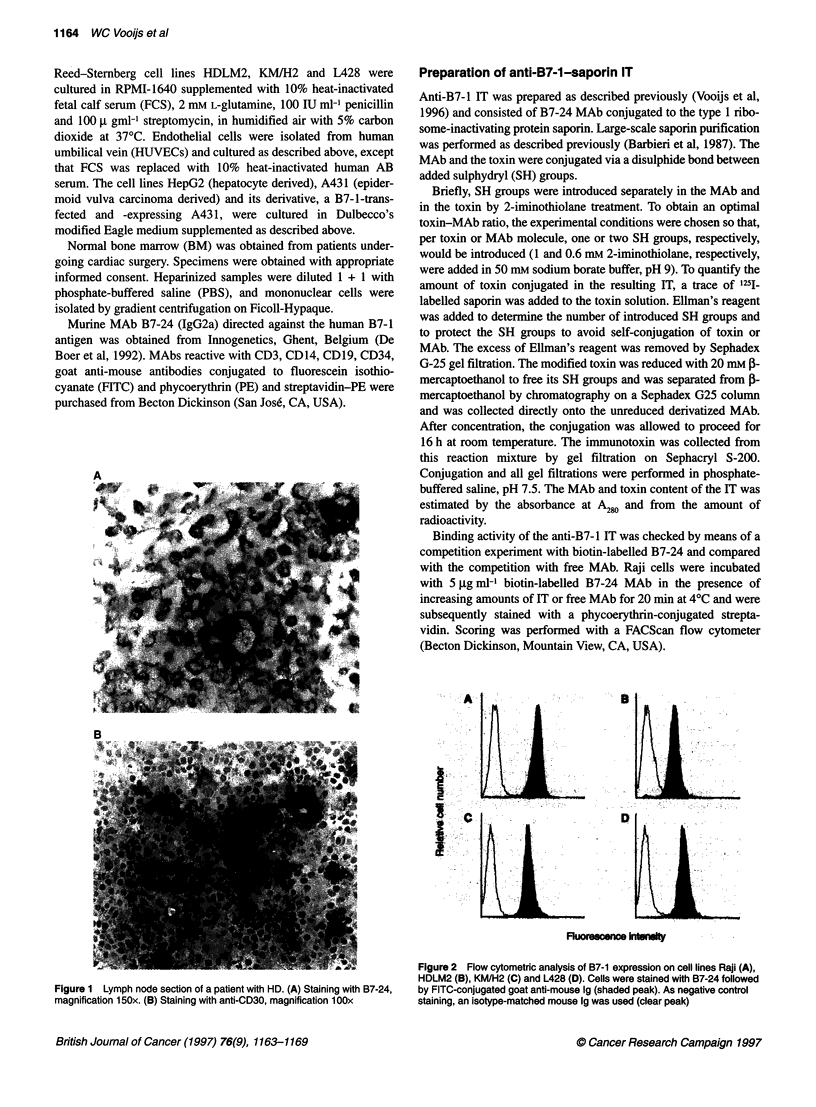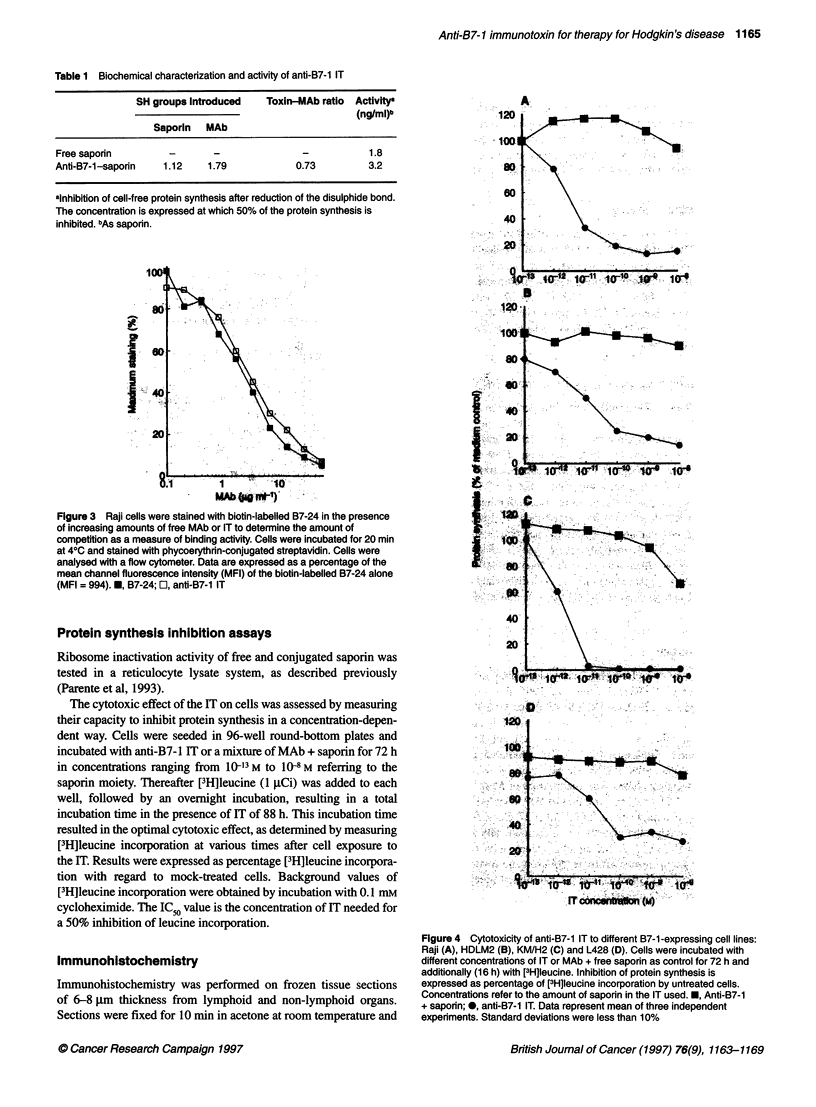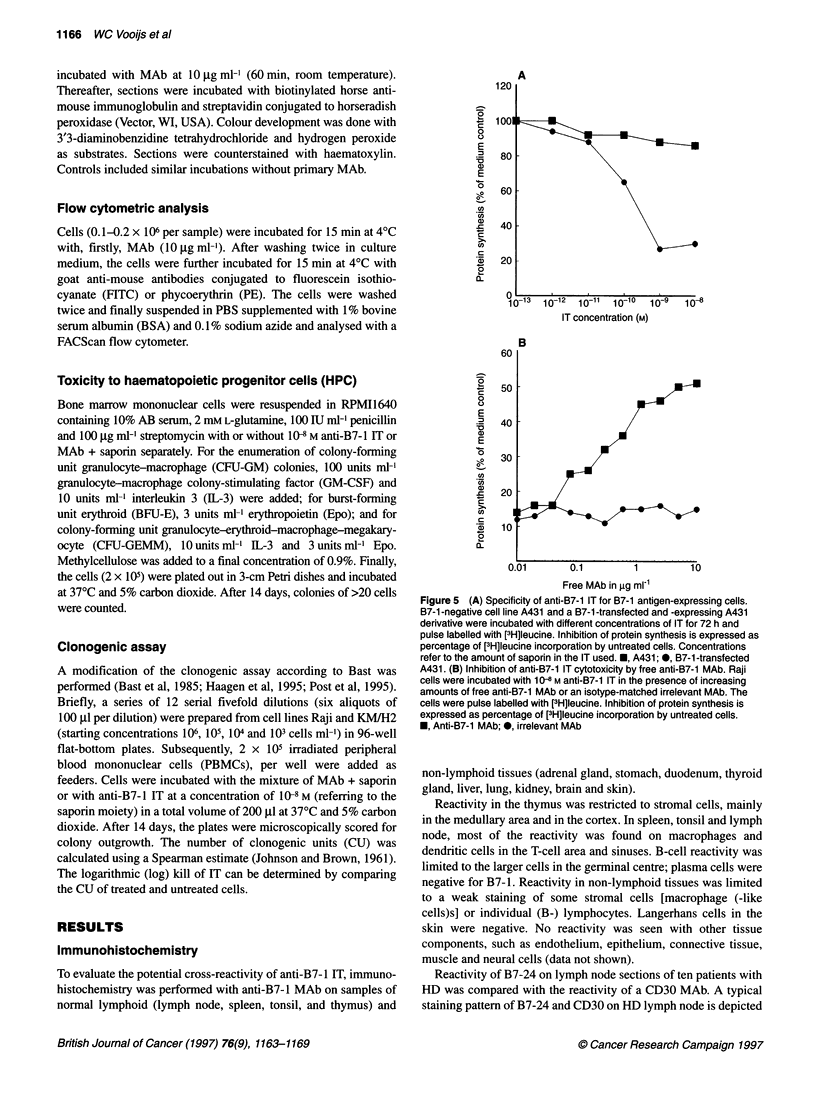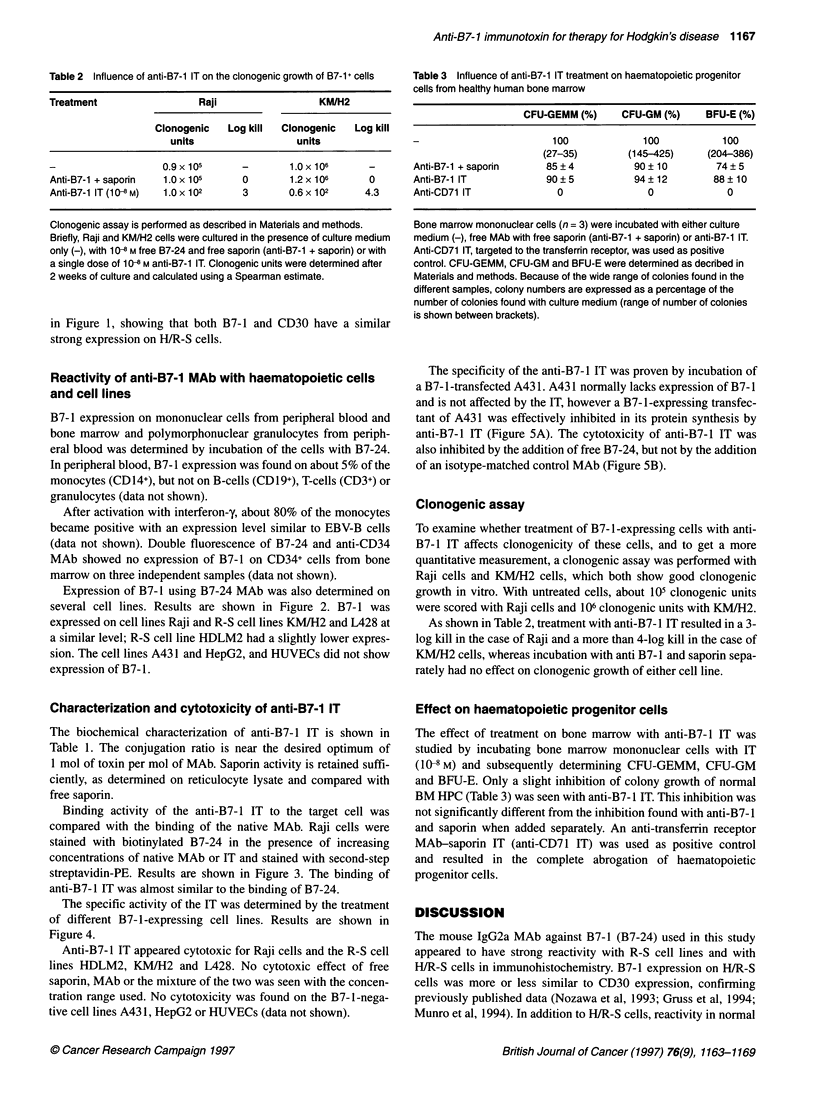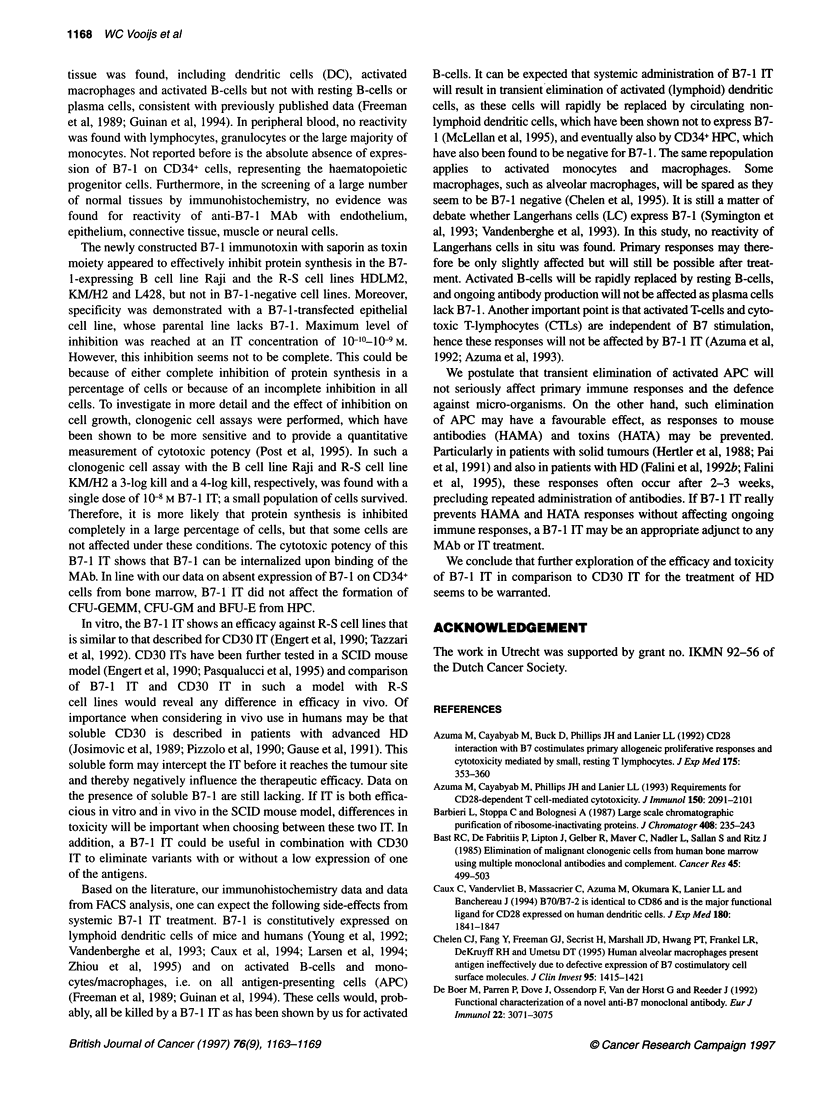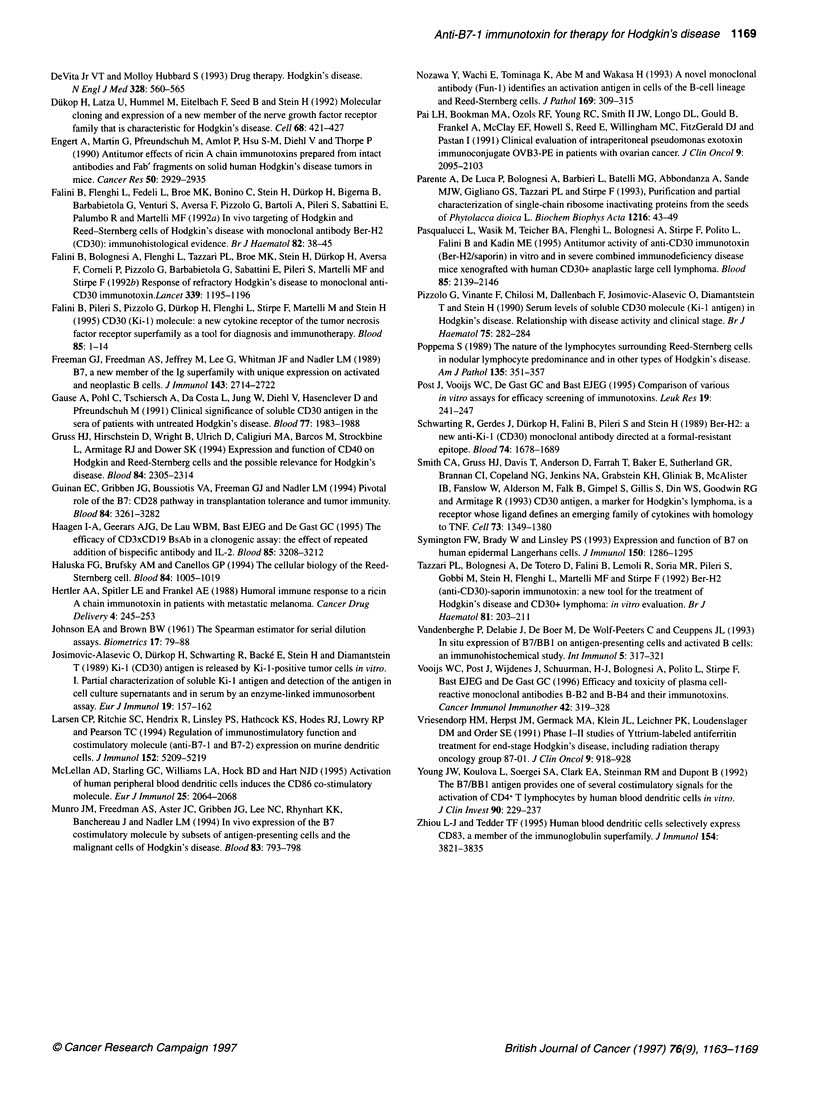# B7-1 (CD80) as target for immunotoxin therapy for Hodgkin's disease.

**DOI:** 10.1038/bjc.1997.528

**Published:** 1997

**Authors:** W. C. Vooijs, H. G. Otten, M. van Vliet, A. J. van Dijk, R. A. de Weger, M. de Boer, H. Bohlen, A. Bolognesi, L. Polito, G. C. de Gast

**Affiliations:** Department of Immuno-haematology, University Hospital Utrecht, The Netherlands.

## Abstract

**Images:**


British Journal of Cancer (1997) 76(9), 1163–1169
© 1997 Cancer Research Campaign

### B7-1 (CD80) as target for immunotoxin therapy for Hodgkin's disease

**WC Vooijs[1], HG Otten[1], M van Vliet[1], AJG van Dijk[1], RA de Weger[2], M de Boer[3], H Bohlen[4], A Bolognesi[5], L Polito[5] and GC de Gast[1]**

Departments of [1]Immuno-haematology and [2]Pathology, University Hospital Utrecht, Utrecht, The Netherlands; [3]Pangenetics, Heemskerk, The Netherlands; [4]Department of Haematology, University Hospital Cologne, Cologne, Germany; [5]Department of Experimental Pathology, University of Bologna, Bologna, Italy

**Summary** In this preclinical study, the potential applicability of an anti-B7-1 immunotoxin (IT) for the treatment of Hodgkin's disease (HD) was investigated. Immunohistochemical analysis demonstrated strong expression of B7-1 on Hodgkin and Reed–Sternberg (R-S) cells and clear expression on dendritic cells, macrophages and some B-cells in tissues, but not on other tissue cells. Flow cytometric analysis demonstrated that B7-1 was expressed on a few monocytes, but not on CD34$^+$ cells from bone marrow, resting T- or B-cells from peripheral blood or epithelial and endothelial cell lines. An anti-B7-1 immunotoxin containing the anti-B7-1 monoclonal antibody (MAb) B7-24 and saporin as toxin moiety was constructed and showed an affinity similar to that shown by the native MAb. It exhibited strong cytotoxicity against the B7-1$^+$ B-cell line Raji ($IC_{50}$ $10^{-11}$ M), R-S cell lines HDLM2, KM/H2 and L428 and also against a B7-1-transfected epithelial cell line, A431, whose parental line lacks expression of B7-1. In clonogenic assays with Raji cells or KM/H2 cells, a 3- or 4-log kill, respectively, was observed. No cytotoxicity was found against the B7-1$^-$ epithelial and endothelial cell lines or against haematopoietic progenitor cells. In conclusion, an anti-B7-1 immunotoxin was developed that had good cytotoxicity against R-S cell lines and that may be used in the elimination of R-S cells in vivo. A concomitant elimination of activated antigen-presenting cells may avoid development of antitoxin and anti-mouse Ig responses and allow repeated administration.

**Keywords:** B7-1; CD80; Hodgkin's disease; immunotoxin; saporin

Hodgkin's disease (HD) comprises a group of malignant lymphomas of mixed cell type that share clinical and pathological features. Typically, there is the presence of mononucleated Hodgkin cells and multinucleated Reed–Sternberg cells (H/R-S) surrounded by a mixture of activated but non-malignant lymphocytes, histiocytes and eosinophils (Poppema, 1989; Haluska et al, 1994). In HD, the constitutional 'B' symptoms such as fever, night sweats, generalized itching and weight loss, and other clinical features, such as eosinophilia, acute-phase reactants, thrombocytosis and sclerosis of HD-involved tissues, are very likely caused by an unbalanced production of cytokines by these H/R-S cells.

HD is a tumour that is highly responsive to both chemotherapy and radiotherapy (DeVita and Molloy Hubbard, 1993). Most patients with early-stage disease can be cured by single-modality treatment. Advanced HD is generally treated with combination chemotherapy with good results. However, about 20% of patients will eventually suffer from a drug-resistant relapse of their disease. For these patients, and for patients with primary resistant HD, other treatment strategies are warranted. A promising approach is immunotherapy using monoclonal antibodies (MAbs) to deliver therapeutically active agents to the cancer site. As H/R-S cells are believed to be the malignant cells in HD, these cells seem to be the best target for such immunotherapy. The antigenic make-up of H/R-S cells include several well-expressed surface antigens: CD15, CD30, CD40, IL-2R (CD25), transferrin receptor (CD71), B7-1 (CD80) and B7-2 (CD86) (Schwarting et al, 1989; Dürkop et al, 1992; Falini et al, 1992*a*; Nozawa et al, 1993; Smith et al, 1993; Gruss et al, 1994; Munro et al, 1994; Falini et al, 1995), to which a therapeutic agent could be targeted. The active moiety coupled to a MAb may consist of a radio isotope (radioimmunoconjugate) (Vriesendorp et al, 1991) or a protein synthesis-inactivating toxin (immunotoxin, IT) (Falini et al, 1992*b*). As treatment modality for HD, immunotoxins may be preferred to radioimmunoconjugates, as the latter are less well targeted to a particular cell type, and other cells around the H/R-S cells will be damaged. Moreover, patients have often been treated with radiotherapy in an early stage of their disease, which may result in radioresistance. The efficacy of an IT depends largely on the choice of the target antigen. As ITs display their toxic action only inside the cell, efficacy of an IT depends, in addition to the level of antigen expression, on internalization and intracellular routing of the IT. Furthermore, not only reactivity of the MAb to the neoplastic cells should be considered but also reactivity to other cells and tissues, as serious problems may arise from using broadly reactive MAbs because of destruction of healthy tissues (Pai et al, 1991).

In this study, we have evaluated whether an anti-B7-1 IT could be a possible HD treatment, taking into account efficacy and toxicity.

*Received 23 January 1997*
*Revised 7 April 1997*
*Accepted 11 April 1997*

*Correspondence to:* WC Vooijs, Department of Haematology, HP F03.722, University Hospital Utrecht, PO Box 85500, 3508 GA Utrecht, The Netherlands

### MATERIALS AND METHODS

### Cell culture and MAbs

Cells of the Burkitt lymphoma-derived B-cell line Raji and the

Reed–Sternberg cell lines HDLM2, KM/H2 and L428 were cultured in RPMI-1640 supplemented with 10% heat-inactivated fetal calf serum (FCS), 2 mM L-glutamine, 100 IU $ml^{-1}$ penicillin and 100 μ $gml^{-1}$ streptomycin, in humidified air with 5% carbon dioxide at 37°C. Endothelial cells were isolated from human umbilical vein (HUVECs) and cultured as described above, except that FCS was replaced with 10% heat-inactivated human AB serum. The cell lines HepG2 (hepatocyte derived), A431 (epidermoid vulva carcinoma derived) and its derivative, a B7-1-transfected and -expressing A431, were cultured in Dulbecco's modified Eagle medium supplemented as described above.

Normal bone marrow (BM) was obtained from patients undergoing cardiac surgery. Specimens were obtained with appropriate informed consent. Heparinized samples were diluted 1 + 1 with phosphate-buffered saline (PBS), and mononuclear cells were isolated by gradient centrifugation on Ficoll-Hypaque.

Murine MAb B7-24 (IgG2a) directed against the human B7-1 antigen was obtained from Innogenetics, Ghent, Belgium (De Boer et al, 1992). MAbs reactive with CD3, CD14, CD19, CD34, goat anti-mouse antibodies conjugated to fluorescein isothiocyanate (FITC) and phycoerythrin (PE) and streptavidin–PE were purchased from Becton Dickinson (San José, CA, USA).

**Figure 1** Lymph node section of a patient with HD. (**A**) Staining with B7-24, magnification 150×. (**B**) Staining with anti-CD30, magnification 100×

### Preparation of anti-B7-1–saporin IT

Anti-B7-1 IT was prepared as described previously (Vooijs et al, 1996) and consisted of B7-24 MAb conjugated to the type 1 ribosome-inactivating protein saporin. Large-scale saporin purification was performed as described previously (Barbieri et al, 1987). The MAb and the toxin were conjugated via a disulphide bond between added sulphydryl (SH) groups.

Briefly, SH groups were introduced separately in the MAb and in the toxin by 2-iminothiolane treatment. To obtain an optimal toxin–MAb ratio, the experimental conditions were chosen so that, per toxin or MAb molecule, one or two SH groups, respectively, would be introduced (1 and 0.6 mM 2-iminothiolane, respectively, were added in 50 mM sodium borate buffer, pH 9). To quantify the amount of toxin conjugated in the resulting IT, a trace of $^{125}I$-labelled saporin was added to the toxin solution. Ellman's reagent was added to determine the number of introduced SH groups and to protect the SH groups to avoid self-conjugation of toxin or MAb. The excess of Ellman's reagent was removed by Sephadex G-25 gel filtration. The modified toxin was reduced with 20 mM β-mercaptoethanol to free its SH groups and was separated from β-mercaptoethanol by chromatography on a Sephadex G25 column and was collected directly onto the unreduced derivatized MAb. After concentration, the conjugation was allowed to proceed for 16 h at room temperature. The immunotoxin was collected from this reaction mixture by gel filtration on Sephacryl S-200. Conjugation and all gel filtrations were performed in phosphate-buffered saline, pH 7.5. The MAb and toxin content of the IT was estimated by the absorbance at $A_{280}$ and from the amount of radioactivity.

Binding activity of the anti-B7-1 IT was checked by means of a competition experiment with biotin-labelled B7-24 and compared with the competition with free MAb. Raji cells were incubated with 5 μg $ml^{-1}$ biotin-labelled B7-24 MAb in the presence of increasing amounts of IT or free MAb for 20 min at 4°C and were subsequently stained with a phycoerythrin-conjugated streptavidin. Scoring was performed with a FACScan flow cytometer (Becton Dickinson, Mountain View, CA, USA).

**Figure 2** Flow cytometric analysis of B7-1 expression on cell lines Raji (**A**), HDLM2 (**B**), KM/H2 (**C**) and L428 (**D**). Cells were stained with B7-24 followed by FITC-conjugated goat anti-mouse Ig (shaded peak). As negative control staining, an isotype-matched mouse Ig was used (clear peak)

© Cancer Research Campaign 1997

**Table 1** Biochemical characterization and activity of anti-B7-1 IT

| | SH groups introduced | | Toxin–MAb ratio | Activity[a] (ng/ml)[b] |
|---|---|---|---|---|
| | Saporin | MAb | | |
| Free saporin | – | – | – | 1.8 |
| Anti-B7-1–saporin | 1.12 | 1.79 | 0.73 | 3.2 |

[a]Inhibition of cell-free protein synthesis after reduction of the disulphide bond. The concentration is expressed at which 50% of the protein synthesis is inhibited. [b]As saporin.

**Figure 3** Raji cells were stained with biotin-labelled B7-24 in the presence of increasing amounts of free MAb or IT to determine the amount of competition as a measure of binding activity. Cells were incubated for 20 min at 4°C and stained with phycoerythrin-conjugated streptavidin. Cells were analysed with a flow cytometer. Data are expressed as a percentage of the mean channel fluorescence intensity (MFI) of the biotin-labelled B7-24 alone (MFI = 994). ■, B7-24; □, anti-B7-1 IT

### Protein synthesis inhibition assays

Ribosome inactivation activity of free and conjugated saporin was tested in a reticulocyte lysate system, as described previously (Parente et al, 1993).

The cytotoxic effect of the IT on cells was assessed by measuring their capacity to inhibit protein synthesis in a concentration-dependent way. Cells were seeded in 96-well round-bottom plates and incubated with anti-B7-1 IT or a mixture of MAb + saporin for 72 h in concentrations ranging from $10^{-13}$ M to $10^{-8}$ M referring to the saporin moiety. Thereafter [$^3$H]leucine (1 μCi) was added to each well, followed by an overnight incubation, resulting in a total incubation time in the presence of IT of 88 h. This incubation time resulted in the optimal cytotoxic effect, as determined by measuring [$^3$H]leucine incorporation at various times after cell exposure to the IT. Results were expressed as percentage [$^3$H]leucine incorporation with regard to mock-treated cells. Background values of [$^3$H]leucine incorporation were obtained by incubation with 0.1 mM cycloheximide. The $IC_{50}$ value is the concentration of IT needed for a 50% inhibition of leucine incorporation.

### Immunohistochemistry

Immunohistochemistry was performed on frozen tissue sections of 6–8 μm thickness from lymphoid and non-lymphoid organs. Sections were fixed for 10 min in acetone at room temperature and

**Figure 4** Cytotoxicity of anti-B7-1 IT to different B7-1-expressing cell lines: Raji (**A**), HDLM2 (**B**), KM/H2 (**C**) and L428 (**D**). Cells were incubated with different concentrations of IT or MAb + free saporin as control for 72 h and additionally (16 h) with [$^3$H]leucine. Inhibition of protein synthesis is expressed as percentage of [$^3$H]leucine incorporation by untreated cells. Concentrations refer to the amount of saporin in the IT used. ■, Anti-B7-1 + saporin; ●, anti-B7-1 IT. Data represent mean of three independent experiments. Standard deviations were less than 10%

© Cancer Research Campaign 1997

incubated with MAb at 10 µg ml$^{-1}$ (60 min, room temperature). Thereafter, sections were incubated with biotinylated horse anti-mouse immunoglobulin and streptavidin conjugated to horseradish peroxidase (Vector, WI, USA). Colour development was done with 3′3-diaminobenzidine tetrahydrochloride and hydrogen peroxide as substrates. Sections were counterstained with haematoxylin. Controls included similar incubations without primary MAb.

### Flow cytometric analysis

Cells (0.1–0.2 × 10$^{6}$ per sample) were incubated for 15 min at 4°C with, firstly, MAb (10 µg ml$^{-1}$). After washing twice in culture medium, the cells were further incubated for 15 min at 4°C with goat anti-mouse antibodies conjugated to fluorescein isothiocyanate (FITC) or phycoerythrin (PE). The cells were washed twice and finally suspended in PBS supplemented with 1% bovine serum albumin (BSA) and 0.1% sodium azide and analysed with a FACScan flow cytometer.

### Toxicity to haematopoietic progenitor cells (HPC)

Bone marrow mononuclear cells were resuspended in RPMI1640 containing 10% AB serum, 2 mM L-glutamine, 100 IU ml$^{-1}$ penicillin and 100 µg ml$^{-1}$ streptomycin with or without 10$^{-8}$ M anti-B7-1 IT or MAb + saporin separately. For the enumeration of colony-forming unit granulocyte–macrophage (CFU-GM) colonies, 100 units ml$^{-1}$ granulocyte–macrophage colony-stimulating factor (GM-CSF) and 10 units ml$^{-1}$ interleukin 3 (IL-3) were added; for burst-forming unit erythroid (BFU-E), 3 units ml$^{-1}$ erythropoietin (Epo); and for colony-forming unit granulocyte–erythroid–macrophage–megakaryocyte (CFU-GEMM), 10 units ml$^{-1}$ IL-3 and 3 units ml$^{-1}$ Epo. Methylcellulose was added to a final concentration of 0.9%. Finally, the cells (2 × 10$^{5}$) were plated out in 3-cm Petri dishes and incubated at 37°C and 5% carbon dioxide. After 14 days, colonies of >20 cells were counted.

### Clonogenic assay

A modification of the clonogenic assay according to Bast was performed (Bast et al, 1985; Haagen et al, 1995; Post et al, 1995). Briefly, a series of 12 serial fivefold dilutions (six aliquots of 100 µl per dilution) were prepared from cell lines Raji and KM/H2 (starting concentrations 10$^{6}$, 10$^{5}$, 10$^{4}$ and 10$^{3}$ cells ml$^{-1}$) in 96-well flat-bottom plates. Subsequently, 2 × 10$^{5}$ irradiated peripheral blood mononuclear cells (PBMCs), per well were added as feeders. Cells were incubated with the mixture of MAb + saporin or with anti-B7-1 IT at a concentration of 10$^{-8}$ M (referring to the saporin moiety) in a total volume of 200 µl at 37°C and 5% carbon dioxide. After 14 days, the plates were microscopically scored for colony outgrowth. The number of clonogenic units (CU) was calculated using a Spearman estimate (Johnson and Brown, 1961). The logarithmic (log) kill of IT can be determined by comparing the CU of treated and untreated cells.

### RESULTS

### Immunohistochemistry

To evaluate the potential cross-reactivity of anti-B7-1 IT, immunohistochemistry was performed with anti-B7-1 MAb on samples of normal lymphoid (lymph node, spleen, tonsil, and thymus) and

**Figure 5** (**A**) Specificity of anti-B7-1 IT for B7-1 antigen-expressing cells. B7-1-negative cell line A431 and a B7-1-transfected and -expressing A431 derivative were incubated with different concentrations of IT for 72 h and pulse labelled with [$^{3}$H]leucine. Inhibition of protein synthesis is expressed as percentage of [$^{3}$H]leucine incorporation by untreated cells. Concentrations refer to the amount of saporin in the IT used. ■, A431; ●, B7-1-transfected A431. (**B**) Inhibition of anti-B7-1 IT cytotoxicity by free anti-B7-1 MAb. Raji cells were incubated with 10$^{-8}$ M anti-B7-1 IT in the presence of increasing amounts of free anti-B7-1 MAb or an isotype-matched irrelevant MAb. The cells were pulse labelled with [$^{3}$H]leucine. Inhibition of protein synthesis is expressed as percentage of [$^{3}$H]leucine incorporation by untreated cells. ■, Anti-B7-1 MAb; ●, irrelevant MAb non-lymphoid tissues (adrenal gland, stomach, duodenum, thyroid gland, liver, lung, kidney, brain and skin).

Reactivity in the thymus was restricted to stromal cells, mainly in the medullary area and in the cortex. In spleen, tonsil and lymph node, most of the reactivity was found on macrophages and dendritic cells in the T-cell area and sinuses. B-cell reactivity was limited to the larger cells in the germinal centre; plasma cells were negative for B7-1. Reactivity in non-lymphoid tissues was limited to a weak staining of some stromal cells [macrophage (-like cells)s] or individual (B-) lymphocytes. Langerhans cells in the skin were negative. No reactivity was seen with other tissue components, such as endothelium, epithelium, connective tissue, muscle and neural cells (data not shown).

Reactivity of B7-24 on lymph node sections of ten patients with HD was compared with the reactivity of a CD30 MAb. A typical staining pattern of B7-24 and CD30 on HD lymph node is depicted

© Cancer Research Campaign 1997

**Table 2** Influence of anti-B7-1 IT on the clonogenic growth of B7-1+ cells

| Treatment | Raji | | KM/H2 | |
|---|---|---|---|---|
| | Clonogenic units | Log kill | Clonogenic units | Log kill |
| – | $0.9 \times 10^5$ | – | $1.0 \times 10^6$ | – |
| Anti-B7-1 + saporin | $1.0 \times 10^5$ | 0 | $1.2 \times 10^6$ | 0 |
| Anti-B7-1 IT ($10^{-8}$ M) | $1.0 \times 10^2$ | 3 | $0.6 \times 10^2$ | 4.3 |

Clonogenic assay is performed as described in Materials and methods. Briefly, Raji and KM/H2 cells were cultured in the presence of culture medium only (–), with $10^{-8}$ M free B7-24 and free saporin (anti-B7-1 + saporin) or with a single dose of $10^{-8}$ M anti-B7-1 IT. Clonogenic units were determined after 2 weeks of culture and calculated using a Spearman estimate.

**Table 3** Influence of anti-B7-1 IT treatment on haematopoietic progenitor cells from healthy human bone marrow

| | CFU-GEMM (%) | CFU-GM (%) | BFU-E (%) |
|---|---|---|---|
| – | 100 (27–35) | 100 (145–425) | 100 (204–386) |
| Anti-B7-1 + saporin | 85 ± 4 | 90 ± 10 | 74 ± 5 |
| Anti-B7-1 IT | 90 ± 5 | 94 ± 12 | 88 ± 10 |
| Anti-CD71 IT | 0 | 0 | 0 |

Bone marrow mononuclear cells ($n$ = 3) were incubated with either culture medium (–), free MAb with free saporin (anti-B7-1 + saporin) or anti-B7-1 IT. Anti-CD71 IT, targeted to the transferrin receptor, was used as positive control. CFU-GEMM, CFU-GM and BFU-E were determined as decribed in Materials and methods. Because of the wide range of colonies found in the different samples, colony numbers are expressed as a percentage of the number of colonies found with culture medium (range of number of colonies is shown between brackets).

in Figure 1, showing that both B7-1 and CD30 have a similar strong expression on H/R-S cells.

### Reactivity of anti-B7-1 MAb with haematopoietic cells and cell lines

B7-1 expression on mononuclear cells from peripheral blood and bone marrow and polymorphonuclear granulocytes from peripheral blood was determined by incubation of the cells with B7-24. In peripheral blood, B7-1 expression was found on about 5% of the monocytes (CD14+), but not on B-cells (CD19+), T-cells (CD3+) or granulocytes (data not shown).

After activation with interferon-γ, about 80% of the monocytes became positive with an expression level similar to EBV-B cells (data not shown). Double fluorescence of B7-24 and anti-CD34 MAb showed no expression of B7-1 on CD34+ cells from bone marrow on three independent samples (data not shown).

Expression of B7-1 using B7-24 MAb was also determined on several cell lines. Results are shown in Figure 2. B7-1 was expressed on cell lines Raji and R-S cell lines KM/H2 and L428 at a similar level; R-S cell line HDLM2 had a slightly lower expression. The cell lines A431 and HepG2, and HUVECs did not show expression of B7-1.

### Characterization and cytotoxicity of anti-B7-1 IT

The biochemical characterization of anti-B7-1 IT is shown in Table 1. The conjugation ratio is near the desired optimum of 1 mol of toxin per mol of MAb. Saporin activity is retained sufficiently, as determined on reticulocyte lysate and compared with free saporin.

Binding activity of the anti-B7-1 IT to the target cell was compared with the binding of the native MAb. Raji cells were stained with biotinylated B7-24 in the presence of increasing concentrations of native MAb or IT and stained with second-step streptavidin-PE. Results are shown in Figure 3. The binding of anti-B7-1 IT was almost similar to the binding of B7-24.

The specific activity of the IT was determined by the treatment of different B7-1-expressing cell lines. Results are shown in Figure 4.

Anti-B7-1 IT appeared cytotoxic for Raji cells and the R-S cell lines HDLM2, KM/H2 and L428. No cytotoxic effect of free saporin, MAb or the mixture of the two was seen with the concentration range used. No cytotoxicity was found on the B7-1-negative cell lines A431, HepG2 or HUVECs (data not shown).

The specificity of the anti-B7-1 IT was proven by incubation of a B7-1-transfected A431. A431 normally lacks expression of B7-1 and is not affected by the IT, however a B7-1-expressing transfectant of A431 was effectively inhibited in its protein synthesis by anti-B7-1 IT (Figure 5A). The cytotoxicity of anti-B7-1 IT was also inhibited by the addition of free B7-24, but not by the addition of an isotype-matched control MAb (Figure 5B).

### Clonogenic assay

To examine whether treatment of B7-1-expressing cells with anti-B7-1 IT affects clonogenicity of these cells, and to get a more quantitative measurement, a clonogenic assay was performed with Raji cells and KM/H2 cells, which both show good clonogenic growth in vitro. With untreated cells, about $10^5$ clonogenic units were scored with Raji cells and $10^6$ clonogenic units with KM/H2.

As shown in Table 2, treatment with anti-B7-1 IT resulted in a 3-log kill in the case of Raji and a more than 4-log kill in the case of KM/H2 cells, whereas incubation with anti B7-1 and saporin separately had no effect on clonogenic growth of either cell line.

### Effect on haematopoietic progenitor cells

The effect of treatment on bone marrow with anti-B7-1 IT was studied by incubating bone marrow mononuclear cells with IT ($10^{-8}$ M) and subsequently determining CFU-GEMM, CFU-GM and BFU-E. Only a slight inhibition of colony growth of normal BM HPC (Table 3) was seen with anti-B7-1 IT. This inhibition was not significantly different from the inhibition found with anti-B7-1 and saporin when added separately. An anti-transferrin receptor MAb–saporin IT (anti-CD71 IT) was used as positive control and resulted in the complete abrogation of haematopoietic progenitor cells.

### DISCUSSION

The mouse IgG2a MAb against B7-1 (B7-24) used in this study appeared to have strong reactivity with R-S cell lines and with H/R-S cells in immunohistochemistry. B7-1 expression on H/R-S cells was more or less similar to CD30 expression, confirming previously published data (Nozawa et al, 1993; Gruss et al, 1994; Munro et al, 1994). In addition to H/R-S cells, reactivity in normal

© Cancer Research Campaign 1997

tissue was found, including dendritic cells (DC), activated macrophages and activated B-cells but not with resting B-cells or plasma cells, consistent with previously published data (Freeman et al, 1989; Guinan et al, 1994). In peripheral blood, no reactivity was found with lymphocytes, granulocytes or the large majority of monocytes. Not reported before is the absolute absence of expression of B7-1 on $CD34^+$ cells, representing the haematopoietic progenitor cells. Furthermore, in the screening of a large number of normal tissues by immunohistochemistry, no evidence was found for reactivity of anti-B7-1 MAb with endothelium, epithelium, connective tissue, muscle or neural cells.

The newly constructed B7-1 immunotoxin with saporin as toxin moiety appeared to effectively inhibit protein synthesis in the B7-1-expressing B cell line Raji and the R-S cell lines HDLM2, KM/H2 and L428, but not in B7-1-negative cell lines. Moreover, specificity was demonstrated with a B7-1-transfected epithelial cell line, whose parental line lacks B7-1. Maximum level of inhibition was reached at an IT concentration of $10^{-10}$–$10^{-9}$ M. However, this inhibition seems not to be complete. This could be because of either complete inhibition of protein synthesis in a percentage of cells or because of an incomplete inhibition in all cells. To investigate in more detail and the effect of inhibition on cell growth, clonogenic cell assays were performed, which have been shown to be more sensitive and to provide a quantitative measurement of cytotoxic potency (Post et al, 1995). In such a clonogenic cell assay with the B cell line Raji and R-S cell line KM/H2 a 3-log kill and a 4-log kill, respectively, was found with a single dose of $10^{-8}$ M B7-1 IT; a small population of cells survived. Therefore, it is more likely that protein synthesis is inhibited completely in a large percentage of cells, but that some cells are not affected under these conditions. The cytotoxic potency of this B7-1 IT shows that B7-1 can be internalized upon binding of the MAb. In line with our data on absent expression of B7-1 on $CD34^+$ cells from bone marrow, B7-1 IT did not affect the formation of CFU-GEMM, CFU-GM and BFU-E from HPC.

In vitro, the B7-1 IT shows an efficacy against R-S cell lines that is similar to that described for CD30 IT (Engert et al, 1990; Tazzari et al, 1992). CD30 ITs have been further tested in a SCID mouse model (Engert et al, 1990; Pasqualucci et al, 1995) and comparison of B7-1 IT and CD30 IT in such a model with R-S cell lines would reveal any difference in efficacy in vivo. Of importance when considering in vivo use in humans may be that soluble CD30 is described in patients with advanced HD (Josimovic et al, 1989; Pizzolo et al, 1990; Gause et al, 1991). This soluble form may intercept the IT before it reaches the tumour site and thereby negatively influence the therapeutic efficacy. Data on the presence of soluble B7-1 are still lacking. If IT is both efficacious in vitro and in vivo in the SCID mouse model, differences in toxicity will be important when choosing between these two IT. In addition, a B7-1 IT could be useful in combination with CD30 IT to eliminate variants with or without a low expression of one of the antigens.

Based on the literature, our immunohistochemistry data and data from FACS analysis, one can expect the following side-effects from systemic B7-1 IT treatment. B7-1 is constitutively expressed on lymphoid dendritic cells of mice and humans (Young et al, 1992; Vandenberghe et al, 1993; Caux et al, 1994; Larsen et al, 1994; Zhiou et al, 1995) and on activated B-cells and monocytes/macrophages, i.e. on all antigen-presenting cells (APC) (Freeman et al, 1989; Guinan et al, 1994). These cells would, probably, all be killed by a B7-1 IT as has been shown by us for activated B-cells. It can be expected that systemic administration of B7-1 IT will result in transient elimination of activated (lymphoid) dendritic cells, as these cells will rapidly be replaced by circulating non-lymphoid dendritic cells, which have been shown not to express B7-1 (McLellan et al, 1995), and eventually also by $CD34^+$ HPC, which have also been found to be negative for B7-1. The same repopulation applies to activated monocytes and macrophages. Some macrophages, such as alveolar macrophages, will be spared as they seem to be B7-1 negative (Chelen et al, 1995). It is still a matter of debate whether Langerhans cells (LC) express B7-1 (Symington et al, 1993; Vandenberghe et al, 1993). In this study, no reactivity of Langerhans cells in situ was found. Primary responses may therefore be only slightly affected but will still be possible after treatment. Activated B-cells will be rapidly replaced by resting B-cells, and ongoing antibody production will not be affected as plasma cells lack B7-1. Another important point is that activated T-cells and cytotoxic T-lymphocytes (CTLs) are independent of B7 stimulation, hence these responses will not be affected by B7-1 IT (Azuma et al, 1992; Azuma et al, 1993).

We postulate that transient elimination of activated APC will not seriously affect primary immune responses and the defence against micro-organisms. On the other hand, such elimination of APC may have a favourable effect, as responses to mouse antibodies (HAMA) and toxins (HATA) may be prevented. Particularly in patients with solid tumours (Hertler et al, 1988; Pai et al, 1991) and also in patients with HD (Falini et al, 1992*b*; Falini et al, 1995), these responses often occur after 2–3 weeks, precluding repeated administration of antibodies. If B7-1 IT really prevents HAMA and HATA responses without affecting ongoing immune responses, a B7-1 IT may be an appropriate adjunct to any MAb or IT treatment.

We conclude that further exploration of the efficacy and toxicity of B7-1 IT in comparison to CD30 IT for the treatment of HD seems to be warranted.

### ACKNOWLEDGEMENT

The work in Utrecht was supported by grant no. IKMN 92–56 of the Dutch Cancer Society.

### REFERENCES

Azuma M, Cayabyab M, Buck D, Phillips JH and Lanier LL (1992) CD28 interaction with B7 costimulates primary allogeneic proliferative responses and cytotoxicity mediated by small, resting T lymphocytes. *J Exp Med* **175**: 353–360

Azuma M, Cayabyab M, Phillips JH and Lanier LL (1993) Requirements for CD28-dependent T cell-mediated cytotoxicity. *J Immunol* **150**: 2091–2101

Barbieri L, Stoppa C and Bolognesi A (1987) Large scale chromatographic purification of ribosome-inactivating proteins. *J Chromatogr* **408**: 235–243

Bast RC, De Fabritiis P, Lipton J, Gelber R, Maver C, Nadler L, Sallan S and Ritz J (1985) Elimination of malignant clonogenic cells from human bone marrow using multiple monoclonal antibodies and complement. *Cancer Res* **45**: 499–503

Caux C, Vandervliet B, Massacrier C, Azuma M, Okumara K, Lanier LL and Banchereau J (1994) B70/B7-2 is identical to CD86 and is the major functional ligand for CD28 expressed on human dendritic cells. *J Exp Med* **180**: 1841–1847

Chelen CJ, Fang Y, Freeman GJ, Secrist H, Marshall JD, Hwang PT, Frankel LR, DeKruyff RH and Umetsu DT (1995) Human alveolar macrophages present antigen ineffectively due to defective expression of B7 costimulatory cell surface molecules. *J Clin Invest* **95**: 1415–1421

De Boer M, Parren P, Dove J, Ossendorp F, Van der Horst G and Reeder J (1992) Functional characterization of a novel anti-B7 monoclonal antibody. *Eur J Immunol* **22**: 3071–3075

© Cancer Research Campaign 1997

DeVita Jr VT and Molloy Hubbard S (1993) Drug therapy. Hodgkin's disease. *N Engl J Med* **328**: 560–565

Dükop H, Latza U, Hummel M, Eitelbach F, Seed B and Stein H (1992) Molecular cloning and expression of a new member of the nerve growth factor receptor family that is characteristic for Hodgkin's disease. *Cell* **68**: 421–427

Engert A, Martin G, Pfreundschuh M, Amlot P, Hsu S-M, Diehl V and Thorpe P (1990) Antitumor effects of ricin A chain immunotoxins prepared from intact antibodies and Fab′ fragments on solid human Hodgkin's disease tumors in mice. *Cancer Res* **50**: 2929–2935

Falini B, Flenghi L, Fedeli L, Broe MK, Bonino C, Stein H, Dürkop H, Bigerna B, Barbabietola G, Venturi S, Aversa F, Pizzolo G, Bartoli A, Pileri S, Sabattini E, Palumbo R and Martelli MF (1992*a*) In vivo targeting of Hodgkin and Reed–Sternberg cells of Hodgkin's disease with monoclonal antibody Ber-H2 (CD30): immunohistological evidence. *Br J Haematol* **82**: 38–45

Falini B, Bolognesi A, Flenghi L, Tazzari PL, Broe MK, Stein H, Dürkop H, Aversa F, Corneli P, Pizzolo G, Barbabietola G, Sabattini E, Pileri S, Martelli MF and Stirpe F (1992*b*) Response of refractory Hodgkin's disease to monoclonal anti-CD30 immunotoxin.*Lancet* **339**: 1195–1196

Falini B, Pileri S, Pizzolo G, Dürkop H, Flenghi L, Stirpe F, Martelli M and Stein H (1995) CD30 (Ki-1) molecule: a new cytokine receptor of the tumor necrosis factor receptor superfamily as a tool for diagnosis and immunotherapy. *Blood* **85**: 1–14

Freeman GJ, Freedman AS, Jeffrey M, Lee G, Whitman JF and Nadler LM (1989) B7, a new member of the Ig superfamily with unique expression on activated and neoplastic B cells. *J Immunol* **143**: 2714–2722

Gause A, Pohl C, Tschiersch A, Da Costa L, Jung W, Diehl V, Hasenclever D and Pfreundschuh M (1991) Clinical significance of soluble CD30 antigen in the sera of patients with untreated Hodgkin's disease. *Blood* **77**: 1983–1988

Gruss HJ, Hirschstein D, Wright B, Ulrich D, Caligiuri MA, Barcos M, Strockbine L, Armitage RJ and Dower SK (1994) Expression and function of CD40 on Hodgkin and Reed-Sternberg cells and the possible relevance for Hodgkin's disease. *Blood* **84**: 2305–2314

Guinan EC, Gribben JG, Boussiotis VA, Freeman GJ and Nadler LM (1994) Pivotal role of the B7: CD28 pathway in transplantation tolerance and tumor immunity. *Blood* **84**: 3261–3282

Haagen I-A, Geerars AJG, De Lau WBM, Bast EJEG and De Gast GC (1995) The efficacy of CD3xCD19 BsAb in a clonogenic assay: the effect of repeated addition of bispecific antibody and IL-2. *Blood* **85**: 3208–3212

Haluska FG, Brufsky AM and Canellos GP (1994) The cellular biology of the Reed-Sternberg cell. *Blood* **84**: 1005–1019

Hertler AA, Spitler LE and Frankel AE (1988) Humoral immune response to a ricin A chain immunotoxin in patients with metastatic melanoma. *Cancer Drug Delivery* **4**: 245–253

Johnson EA and Brown BW (1961) The Spearman estimator for serial dilution assays. *Biometrics* **17**: 79–88

Josimovic-Alasevic O, Dürkop H, Schwarting R, Backé E, Stein H and Diamantstein T (1989) Ki-1 (CD30) antigen is released by Ki-1-positive tumor cells *in vitro*. I. Partial characterization of soluble Ki-1 antigen and detection of the antigen in cell culture supernatants and in serum by an enzyme-linked immunosorbent assay. *Eur J Immunol* **19**: 157–162

Larsen CP, Ritchie SC, Hendrix R, Linsley PS, Hathcock KS, Hodes RJ, Lowry RP and Pearson TC (1994) Regulation of immunostimulatory function and costimulatory molecule (anti-B7-1 and B7-2) expression on murine dendritic cells. *J Immunol* **152**: 5209–5219

McLellan AD, Starling GC, Williams LA, Hock BD and Hart NJD (1995) Activation of human peripheral blood dendritic cells induces the CD86 co-stimulatory molecule. *Eur J Immunol* **25**: 2064–2068

Munro JM, Freedman AS, Aster JC, Gribben JG, Lee NC, Rhynhart KK, Banchereau J and Nadler LM (1994) In vivo expression of the B7 costimulatory molecule by subsets of antigen-presenting cells and the malignant cells of Hodgkin's disease. *Blood* **83**: 793–798

Nozawa Y, Wachi E, Tominaga K, Abe M and Wakasa H (1993) A novel monoclonal antibody (Fun-1) identifies an activation antigen in cells of the B-cell lineage and Reed-Sternberg cells. *J Pathol* **169**: 309–315

Pai LH, Bookman MA, Ozols RF, Young RC, Smith II JW, Longo DL, Gould B, Frankel A, McClay EF, Howell S, Reed E, Willingham MC, FitzGerald DJ and Pastan I (1991) Clinical evaluation of intraperitoneal pseudomonas exotoxin immunoconjugate OVB3-PE in patients with ovarian cancer. *J Clin Oncol* **9**: 2095–2103

Parente A, De Luca P, Bolognesi A, Barbieri L, Batelli MG, Abbondanza A, Sande MJW, Gigliano GS, Tazzari PL and Stirpe F (1993), Purification and partial characterization of single-chain ribosome inactivating proteins from the seeds of *Phytolacca dioica* L. *Biochem Biophys Acta* **1216**: 43–49

Pasqualucci L, Wasik M, Teicher BA, Flenghi L, Bolognesi A, Stirpe F, Polito L, Falini B and Kadin ME (1995) Antitumor activity of anti-CD30 immunotoxin (Ber-H2/saporin) in vitro and in severe combined immunodeficiency disease mice xenografted with human CD30+ anaplastic large cell lymphoma. *Blood* **85**: 2139–2146

Pizzolo G, Vinante F, Chilosi M, Dallenbach F, Josimovic-Alasevic O, Diamantstein T and Stein H (1990) Serum levels of soluble CD30 molecule (Ki-1 antigen) in Hodgkin's disease. Relationship with disease activity and clinical stage. *Br J Haematol* **75**: 282–284

Poppema S (1989) The nature of the lymphocytes surrounding Reed-Sternberg cells in nodular lymphocyte predominance and in other types of Hodgkin's disease. *Am J Pathol* **135**: 351–357

Post J, Vooijs WC, De Gast GC and Bast EJEG (1995) Comparison of various *in vitro* assays for efficacy screening of immunotoxins. *Leuk Res* **19**: 241–247

Schwarting R, Gerdes J, Dürkop H, Falini B, Pileri S and Stein H (1989) Ber-H2: a new anti-Ki-1 (CD30) monoclonal antibody directed at a formal-resistant epitope. *Blood* **74**: 1678–1689

Smith CA, Gruss HJ, Davis T, Anderson D, Farrah T, Baker E, Sutherland GR, Brannan CI, Copeland NG, Jenkins NA, Grabstein KH, Gliniak B, McAlister IB, Fanslow W, Alderson M, Falk B, Gimpel S, Gillis S, Din WS, Goodwin RG and Armitage R (1993) CD30 antigen, a marker for Hodgkin's lymphoma, is a receptor whose ligand defines an emerging family of cytokines with homology to TNF. *Cell* **73**: 1349–1380

Symington FW, Brady W and Linsley PS (1993) Expression and function of B7 on human epidermal Langerhans cells. *J Immunol* **150**: 1286–1295

Tazzari PL, Bolognesi A, De Totero D, Falini B, Lemoli R, Soria MR, Pileri S, Gobbi M, Stein H, Flenghi L, Martelli MF and Stirpe F (1992) Ber-H2 (anti-CD30)-saporin immunotoxin: a new tool for the treatment of Hodgkin's disease and CD30+ lymphoma: *in vitro* evaluation. *Br J Haematol* **81**: 203–211

Vandenberghe P, Delabie J, De Boer M, De Wolf-Peeters C and Ceuppens JL (1993) In situ expression of B7/BB1 on antigen-presenting cells and activated B cells: an immunohistochemical study. *Int Immunol* **5**: 317–321

Vooijs WC, Post J, Wijdenes J, Schuurman, H-J, Bolognesi A, Polito L, Stirpe F, Bast EJEG and De Gast GC (1996) Efficacy and toxicity of plasma cell-reactive monoclonal antibodies B-B2 and B-B4 and their immunotoxins. *Cancer Immunol Immunother* **42**: 319–328

Vriesendorp HM, Herpst JM, Germack MA, Klein JL, Leichner PK, Loudenslager DM and Order SE (1991) Phase I–II studies of Yttrium-labeled antiferritin treatment for end-stage Hodgkin's disease, including radiation therapy oncology group 87-01. *J Clin Oncol* **9**: 918–928

Young JW, Koulova L, Soergei SA, Clark EA, Steinman RM and Dupont B (1992) The B7/BB1 antigen provides one of several costimulatory signals for the activation of CD4+ T lymphocytes by human blood dendritic cells *in vitro*. *J Clin Invest* **90**: 229–237

Zhiou L-J and Tedder TF (1995) Human blood dendritic cells selectively express CD83, a member of the immunoglobulin superfamily. *J Immunol* **154**: 3821–3835

© Cancer Research Campaign 1997